# Heterogeneity revealed by integrated genomic analysis uncovers a molecular switch in malignant uveal melanoma

**DOI:** 10.18632/oncotarget.5637

**Published:** 2015-10-08

**Authors:** Mark J. de Lange, Sake I. van Pelt, Mieke Versluis, Ekaterina S. Jordanova, Wilma G.M. Kroes, Claudia Ruivenkamp, Sjoerd H. van der Burg, Grégorius P.M. Luyten, Thorbald van Hall, Martine J. Jager, Pieter A. van der Velden

**Affiliations:** ^1^ Department of Ophthalmology, LUMC, Leiden, The Netherlands; ^2^ Department of Pathology, LUMC, Leiden, The Netherlands; ^3^ Department of Clinical Genetics Laboratory for Diagnostic Genome Analysis (LDGA), LUMC, Leiden, The Netherlands; ^4^ Department of Clinical Oncology, LUMC, Leiden, The Netherlands

**Keywords:** uveal melanoma, tumor heterogeneity, digital PCR, immune profile, molecular etiology

## Abstract

Gene expression profiles as well as genomic imbalances are correlated with disease progression in uveal melanoma (UM). We integrated expression and genomic profiles to obtain insight into the oncogenic mechanisms in development and progression of UM. We used tumor tissue from 64 enucleated eyes of UM patients for profiling. Mutations and genomic imbalances were quantified with digital PCR to study tumor heterogeneity and molecular pathogenesis. Gene expression analysis divided the UM panel into three classes. Class I presented tumors with a good prognosis and a distinct genomic make up that is characterized by 6p gain. The UM with a bad prognosis were subdivided into class IIa and class IIb. These classes presented similar survival risks but could be distinguished by tumor heterogeneity. Class IIa presented homogeneous tumors while class IIb tumors, on average, contained 30% of non-mutant cells. Tumor heterogeneity coincided with expression of a set of immune genes revealing an extensive immune infiltrate in class IIb tumors. Molecularly, class IIa and IIb presented the same genomic configuration and could only be distinguished by 8q copy number. Moreover, UM establish in the void of the immune privileged eye indicating that in IIb tumors the infiltrate is attracted by the UM. Combined our data show that chromosome 8q contains the locus that causes the immune phentotype of UM. UM thereby provides an unique opportunity to study immune attraction by tumors.

## INTRODUCTION

Uveal melanoma (UM) is a rare ocular neoplasm characterized by GNAQ and GNA11 mutations [1, 2]. Despite changes in treatment, the overall survival rate remains low and patients still die due to metastases which are usually found after the initial diagnosis [3, 4].

For years, monosomy of chromosome 3 has been the most described biomarker that predicts survival in UM patients [5]. Markers on chromosome 3 that underlie this unique subdivision are largely unknown but the chromosome 3 status divides UM genetically into two groups which have a good and a bad prognosis [6]. This finding is supported by clinical and histopathological markers [7]: UM containing epithelioid cells are associated with monosomy 3 and a bad prognosis while UM purely made up of spindle cells are rarely lethal [8, 9]. Larger tumors are correlated with a worse prognosis and tend to have an epithelial/mixed phenotype rather than a spindle cell phenotype. With SNP [10–13], CGH [14–17] and karyotype analysis [18, 19], recurrent chromosomal aberrations were further investigated in UM and shown to be correlated with disease progression. Gain of chromosome 8q and 6p is frequently detected while loss of 1p and 16q loss is less common but is still found to be correlated with prognosis [15, 20, 21]. Although these tumor characteristics can be applied to precisely predict disease outcome, we do not yet understand the sequence of genetic events in the development of UM, nor the relationship between the genetic changes and tumor behavior.

To better understand UM progression and develop targeted therapy, we performed genome-wide gene expression analysis and chromosome analysis of 64 tumors. We integrated expression and structural analysis to obtain insight into the molecular etiology of prognostic groups. We investigated prognostic genes and correlated them to chromosomal aberrations to visualize UM molecularly. Additionally, we validated structural aberrations with digital PCR (dPCR) which revealed the heterogeneity of UM. Cellular heterogeneity provides an insight into the micro environment of UM while detailed molecular heterogeneity is the basis for a molecular progression model, showing that increased copy number of 8q precedes loss of chromosome 3. Moreover, our data show that distinct genetic events give rise to different classes of UM, impacting prognosis and therapy.

## RESULTS

### Expression profiles segregate UM into class I, IIa and IIb

**Figure 1:**
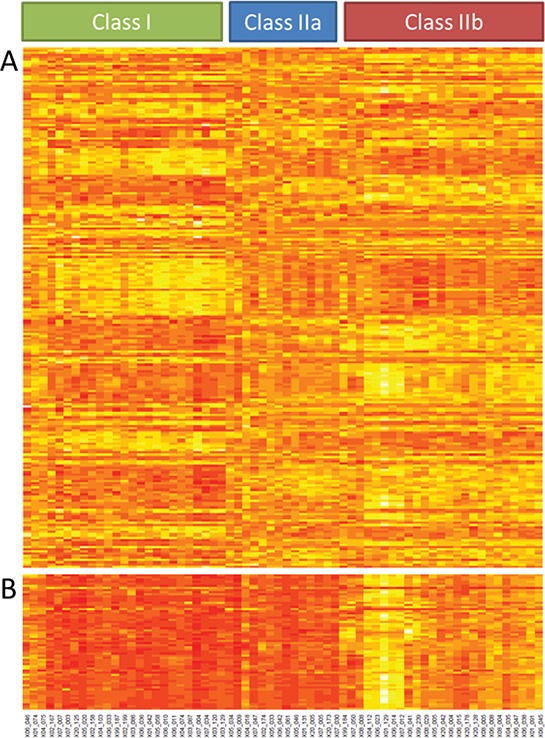
Gene expression analysis Unsupervised clustering of gene expression of 64 UM divides tumors in two classes. Supervised cluster analysis with the 237 most differentially expressed genes resulted in 3 classes because class II is subdivided in class IIa and class IIb **A.** Supervised clustering of the 53 class IIb classifier genes **B.**

Unsupervised cluster analysis of gene expression data of ~16,000 unique genes in 64 primary UM subdivided the tumors into two distinct classes [22]. Class I tumors presented a good prognosis while class II tumors were correlated with a bad prognosis similar to what has been shown previously (Supplementary Figure S1) [6]. Out of the 4,000 differentially expressed genes, 237 genes met the criteria of a Log fold change (LFC) of at least 1.0 and a *p*-value less than 0.05. A total of 132 genes presented a lower expression in class I than in class II, while the remaining 105 displayed a higher expression in class I. When using these 237 differentially expressed genes for cluster analysis, the UM cohort split into 3 clusters. Class I remained the same as with unsupervised analysis, but class II was subdivided into class IIa and IIb (Figure 1A).

Supervised gene expression analysis for IIa and IIb revealed a select panel of genes (*n* = 53) that was differentially expressed between class IIa and IIb (Supplementary Table S2). Among these genes, only two genes showed a higher expression in class IIa compared to IIb while 51 genes showed a higher expression in IIb (Figure 1B). Survival analysis revealed no significant difference between class IIa and IIb (not shown).

### Chromosomal aberrations are specific for UM classes

**Figure 2:**
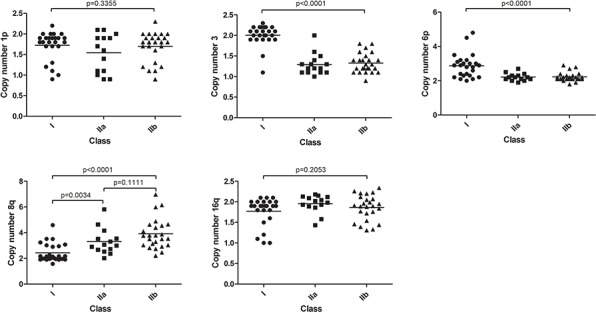
Chromosomal aberrations in expression classes Five recurrent abnormalities distributed over the gene expression classes. Differences between class I and II were mostly seen in chromosome 3 and 6. Gain of chromosome 8q appears to be the best classifier for IIa and IIb subdivision. Non-significant trends were seen in chromosome 1p and 16q.

In order to investigate which genetic mechanisms underlie the subdivision into 3 classes, we investigated genomic aberrations in UM. With SNP analysis, five recurring chromosomal aberrations were detected which were validated with dPCR (Table 1). Loss of chromosome 1p, loss of chromosome 3, gain of chromosome 6p, gain of chromosome 8q and loss of chromosome 16q were common events. The distribution of the chromosomal aberrations (Figure 2) as well as their copy numbers (Supplementary Table S3) were plotted for the three classes showing that the chromosomal aberrations are non-randomly distributed over the three classes.

**Table 1:** Copy number analysis

Class	Tumor no.	FA	Chr 1		Chr 3		Chr 6p		Chr 8q		Chr 16q	
		*GNAQ/11*	*dPCR*	*SNP*	*dPCR*	*SNP*	*dPCR*	*SNP*	*dPCR*	*SNP*	*dPCR*	*SNP*
I	06-046*	35.0**	1.7	2.0	2.0	1.9	2.7	2.6	2.7	2.7	1.4	1.3
I	01-074	48.9	1.2	1.4	2.2	1.9	2.9	2.0	3.5	2.7	2.1	2.3
I	04-075	37.1	1.8	1.7	2.0	1.9	2.8	1.9	3.0	2.9	2.0	2.0
I	02-167	45.1	1.3	1.5	1.5	1.6	2.3	2.1	2.1	2.1	2.0	2.0
I	07-007	36.8	1.7	1.9	1.1	1.0	2.9	2.8	1.9	1.9	2.0	1.8
I	07-003	37.9	1.1	1.0	2.2	2.0	2.2	2.0	2.2	1.9	1.9	1.8
I	20-125	38.5	2.0	1.9	2.2	2.0	4.8	2.1	3.2	2.7	1.9	1.9
I	05-020	34.3	1.0	1.2	1.9	1.9	3.5	3.4	4.6	4.5	1.0	1.1
I	02-158	40.2	2.0	2.0	2.3	2.0	4.5	1.6	2.2	2.0	1.5	1.6
I	04-103	44.8	1.8	1.9	1.9	2.0	2.9	1.3	1.9	2.0	1.9	2.1
I	06-033	46.7	0.9	1.1	2.1	2.1	3.0	3.1	3.0	3.2	1.0	1.0
I	99-187	43.2	1.9	1.9	2.2	2.0	2.2	2.0	2.2	2.0	1.1	1.6
I	02-199	42.2	2.0	1.7	2.0	1.8	2.4	2.2	2.0	1.9	2.0	1.9
I	03-086	53.8	1.9	1.9	2.2	2.0	2.3	2.0	2.0	1.9	2.1	2.0
I	06-036	47.4	1.9	2.0	2.2	2.0	3.2	3.1	2.9	3.1	2.1	1.9
I	01-042	50.0**	1.8	1.8	2.1	2.0	2.1	2.1	1.9	1.9	2.0	2.0
I	05-058	49	1.8	2.0	1.9	2.0	2.8	2.8	1.9	1.9	1.8	1.9
I	06-010	41.2	1.7	1.9	2.0	2.0	2.4	2.2	2.0	2.0	1.9	1.8
I	06-011	43	2.2	2.0	2.1	2.0	3.5	2.9	3.5	3.2	1.2	1.2
I	03-087*	44.4	1.7	1.9	2.0	2.1	2.4	2.1	1.9	2.0	1.6	1.8
I	04-074	48.5	1.8	2.2	1.9	2.0	3.0	2.1	2.0	2.0	1.9	2.0
I	07-004	33.6	1.9	1.9	2.1	2.1	3.2	3.2	2.0	2.0	1.8	1.8
I	07-034	46.7	1.7	2.0	2.0	1.9	3.0	2.8	2.1	2.0	1.9	1.9
I	03-120	52	1.9	1.9	2.0	2.0	3.1	2.8	1.9	1.9	1.9	1.8
I	03-129	29.5	1.9	1.9	1.4	1.5	2.0	2.1	3.1	2.7	1.9	1.6
IIa	05-034	36.4	1.6	1.6	1.6	1.5	2.2	2.2	3.1	3.1	1.6	1.5
IIa	06-009	49	1.1	1.1	1.0	1.1	2.5	2.3	3.0	3.2	1.9	2.1
IIa	04-018	55.4	1.9	2.0	2.0	1.9	2.7	2.0	2.7	2.3	1.9	1.9
IIa	07-047	24.3	0.9	1.0	1.1	1.0	2.0	2.0	2.0	2.0	1.9	1.8
IIa	02-174	47.3	0.9	1.3	1.1	1.4	2.1	2.2	2.7	2.6	2.0	2.1
IIa	05-033	45.7	1.4	1.5	1.2	1.1	2.0	1.9	2.4	2.1	2.2	1.9
IIa	06-042	34	1.9	1.9	1.3	1.1	2.2	2.1	2.9	3.0	2.0	1.9
IIa	05-061	45.2	1.0	1.1	1.1	1.0	2.2	2.0	5.8	5.7	2.1	1.9
IIa	05-046	43.1	1.1	1.1	1.1	1.1	2.1	1.9	3.5	3.7	1.9	1.9
IIa	01-131	35.5	2.1	2.1	1.5	1.5	2.3	2.1	4.6	3.4	1.4	1.6
IIa	20-005	45.6	1.9	2.0	1.2	1.4	2.3	2.1	3.3	2.9	2.1	2.2
IIa	07-005	42.9	1.7	1.9	1.3	1.3	1.9	2.3	4.2	3.9	2.0	1.9
IIa	20-173	43.7	2.0	1.9	1.3	1.3	2.4	2.1	2.5	2.3	2.2	2.3
IIa	07-030	44.5	2.1	2.0	1.2	1.1	2.1	2.0	3.5	3.2	2.1	1.9
IIb	99-184	36.7	1.8	2.0	1.4	1.5	2.1	1.9	4.4	3.3	1.4	1.7
IIb	08-008	39.5**	2.0	1.9	1.2	1.1	2.1	2.0	2.9	2.7	2.2	1.8
IIb	07-050	37.1	1.1	1.2	1.2	1.1	2.1	2.0	4.9	4.9	2.1	2.0
IIb	04-112	44.3	1.7	2.0	1.1	1.3	1.9	2.1	3.5	3.5	1.9	2.0
IIb	06-023	31.5	1.9	2.0	1.4	1.3	2.1	2.1	2.5	2.6	1.9	1.9
IIb	01-129	12.8	1.9	2.0	1.8	1.8	2.1	2.1	2.2	2.1	2.0	2.0
IIb	06-014	19.8	2.0	2.0	1.8	1.6	2.4	2.2	4.7	4.5	1.8	1.6
IIb	07-012	15.8	2.0	1.9	1.7	1.5	2.3	2.0	3.8	3.7	2.2	2.0
IIb	06-041	42	1.8	1.9	1.3	1.2	2.2	2.0	4.6	4.7	1.9	1.8
IIb	99-239	43.4	1.6	1.8	0.9	1.4	1.8	1.9	3.5	3.3	1.3	1.9
IIb	08-029	40.4	1.1	1.3	1.3	1.2	2.1	2.1	3.5	3.2	2.2	1.9
IIb	05-005	21.9	1.7	1.9	1.4	1.3	2.0	2.0	3.1	3.2	1.8	1.8
IIb	20-042	35.9	1.8	1.6	1.6	1.5	2.9	1.9	3.9	3.1	2.3	2.0
IIb	06-004	43.5	1.9	2.1	1.1	1.1	2.1	2.0	3.8	4.2	1.8	1.9
IIb	20-178	27.3	0.9	1.2	1.1	1.3	2.1	2.0	6.0	4.2	1.9	2.1
IIb	06-015	44	1.2	1.3	1.2	1.0	2.3	2.1	3.8	3.9	2.1	1.9
IIb	20-128	39.3	2.3	2.0	1.5	1.5	2.7	1.6	6.2	3.9	1.4	1.7
IIb	08-005	27.4	1.3	1.4	1.2	1.3	2.4	2.0	7.0	5.7	1.3	1.3
IIb	06-008	36.3	1.2	1.1	1.4	1.2	2.2	2.0	3.0	2.9	2.3	1.9
IIb	08-004	33.5**	1.7	1.9	1.2	1.2	2.8	2.8	2.8	2.8	2.0	1.8
IIb	04-035	43	1.8	2.1	1.1	1.3	2.1	2.2	3.0	2.7	2.0	1.9
IIb	06-047	35.8	2.0	1.9	1.4	1.2	2.2	2.0	2.7	2.8	2.1	1.8
IIb	06-038	39.6	2.0	2.1	1.1	1.1	2.2	2.1	3.3	3.4	1.5	1.4
IIb	01-091	44	1.9	2.0	1.2	1.4	2.1	2.1	4.6	3.8	1.5	1.7
IIb	06-045	31.1	1.8	1.8	1.6	1.5	2.2	2.0	4.1	3.8	1.7	1.5

Copy number values of 64 UM measured with both SNP and dPCR analysis. Fractional abundance (FA) denotes the fraction of mutant alleles.

* Chromosome 5 imbalance, *TTC5* copy number used as reference.

** FA values were calculated with copy number analysis.

### Chromosome 3 loss and gain of 6p define tumor class

**Figure 3:**
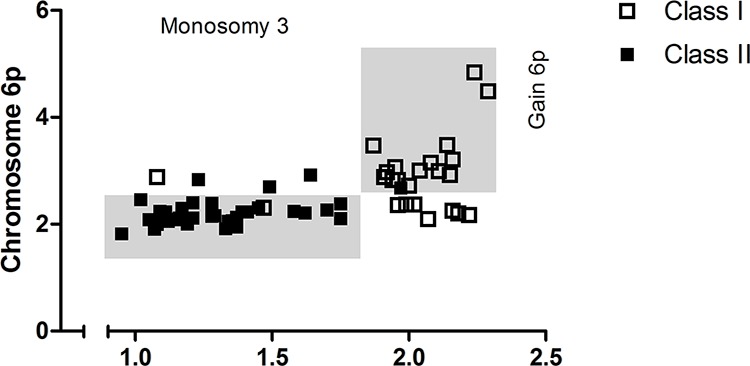
Chromosomal anomalies support UM subdivision Monosomy 3 and 6p gain divided UM in class I and class II. In the mixed tumors 6p copy number was low.

Loss of chromosome 3 was predominantly presented in class II (a/b) tumors and was only detected in 3 out 25 class I tumors (Supplementary Table S3). In contrast, gain of chromosomal part 6p was most prominent in class I tumors. Moreover, co-occurrence of 6p gain and monosomy 3 in UM was rare and 6p copy number was low in these instances, marginally exceeding the thresholds for gain (Figure 3).

### Increased 8q copy number in class IIb tumors

Monosomy 3 and 6p gain molecularly characterize class II and class I tumors, respectively, but do not differentiate class IIa from class IIb tumors. However with analysis of the aberrations of 8q and to a lesser extent 16q this turned out to be possible. Class IIb tumors presented a higher 8q copy number than class IIa tumors (Figure 2). Moreover, the mechanism underlying gain of 8q was different in these two UM classes. Amplification of 8q observed in class IIb was almost exclusively caused by isochromosome formation. The limited gain of 8q that was observed in class IIa tumors was more often due to gain of the entire chromosome [23]. Loss of 16q was not significantly differentially distributed over the three UM classes.

### Functional and genetic annotation

To investigate the link between chromosomal aberrations and differentially expressed genes we analyzed potential genetic and functional correlations of the 237 top differentially expressed genes (Figure 1). Functional annotation showed that 6 terms were significantly overrepresented in the 237 genes (Table 2). These terms concerned the immune system (*n* = 4) and the translation machinery (*n* = 2).

**Table 2:** Functional annotation of classifier genes

Term	Count	Bonferroni	Class
*Antigen processing and presentation*	16	5.8E-11	IIa–IIb
*Immune response*	34	6.7E-09	IIa–IIb
*Translational elongation*	15	1.8E-08	I–II
*Antigen processing and presentation of peptide antigen*	10	3E-08	IIa–IIb
*Antigen processing and presentation of peptide antigen via MHC class I*	7	0.000039	IIa–IIb
*Translation*	19	0.00011	I–II

Functional annotation of the most differentially expressed genes between class I and II. After correction for multiple testing, 6 terms were found to be significantly overrepresented.

Among the 237 genes we identified 151 genes that define the difference between class I and class II in general (Supplementary Table S2). These genes were differentially expressed (LFC > 1 or LFC < −1; *p* < 0.05) between class I and class II but did not significantly differ between class IIa and IIb. Genes on chromosome 3 and 6 were overrepresented with respectively 16 and 12 genes. All of the 16 genes from chromosome 3 showed a lower expression in class II tumors compared to class I tumors corresponding to loss of chromosome 3 in class II. In contrast, the 12 genes on 6p showed either an elevated or a decreased expression in class II. Functional annotation of the differentially-expressed genes showed that the terms all concerned ribosomal and other translational machinery proteins (Table 2).

### The class IIb classifier genes are involved in the immune response

Next the genes differentially-expressed between class IIa and IIb tumors were annotated. In total, 53 genes were significantly differentially expressed (LFC > 1 or < −1; *p* < 0.05) between class IIa and IIb tumors but did not differ between class I and IIa tumors (Supplementary Table S2). There was no clustering of genes to chromosome 8q or 16q. Remarkably, 26 genes on chromosome 6, many of which are involved in the immune response, revealed a significant overrepresentation. Of the 26 genes located on chromosome 6, a significant part is involved in HLA/T-cell reactions. Moreover, many of the genes that define class IIb, appear to be targets of interferon. These data suggest that part of the different gene expression between class IIb and class I and IIa tumors may be due to tumor-resident non-cancer cells.

### Tumor heterogeneity in UM

Based on the assumption that every tumor cell within a mutant UM l either carries a GNAQ or GNA11 mutation we were able to calculate the fractional abundance (FA), which represents the ratio between cancer cells and non-cancer cells (e.g. fibroblasts, immune cells) within the tumor. Four tumors did not present one of the hotspot mutations in GNAQ/11 when analyzed with dPCR and in these cases the monosomy 3 status was used to calculate the FA. This was warranted by the positive correlation between tumor fraction calculated with GNAQ/GNA11 mutation and monosomy 3 (Supplementary Figure S2).

**Figure 4:**
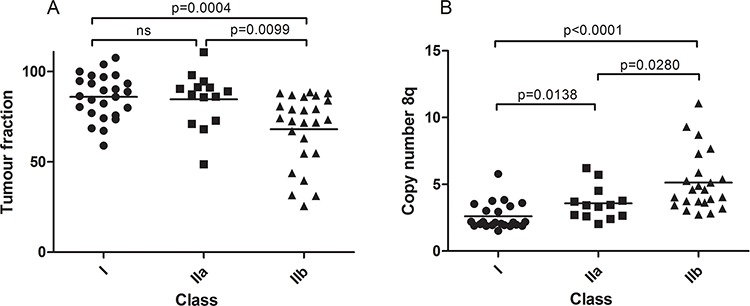
Quantification of tumor heterogeneity Tumor fractions based on GNAQ/11 mutation analyzed with dPCR. Class IIb contains tumors with a significantly lower tumor fraction **A.** After adjusting chromosome 8q copy number for tumor fraction, significant differences were also found between class IIa and class IIb besides the significant differences between class I and class II **B.**

Figure 4A shows the fraction of tumor cells and their distribution over the three classes. No significant difference was found between class I and IIa but a significantly decreased tumor cell percentage was detected in class IIb tumors, sustaining the previous notion that the different chromosome 6 associated gene expression in class IIb tumors may be due to non-tumor cells. As the copy number of the aberrations in this study is the result of admixture of normal cells and tumor cells, we also calculated corrected copy numbers. Fractional abundance of tumor cells was used for a more precise calculation of chromosome 8q copy numbers in the tumor cells (Figure 4B). The mean 8q copy number after adjustment in class I, class IIa and class IIb, was 2.6, 3.5 and 5.0, respectively. For the other aberrations, a dosage effect is not clinically relevant, and therefore we did not calculate adjusted copy numbers.

### Genetic heterogeneity in UM revealed clonal evolution

**Figure 5:**
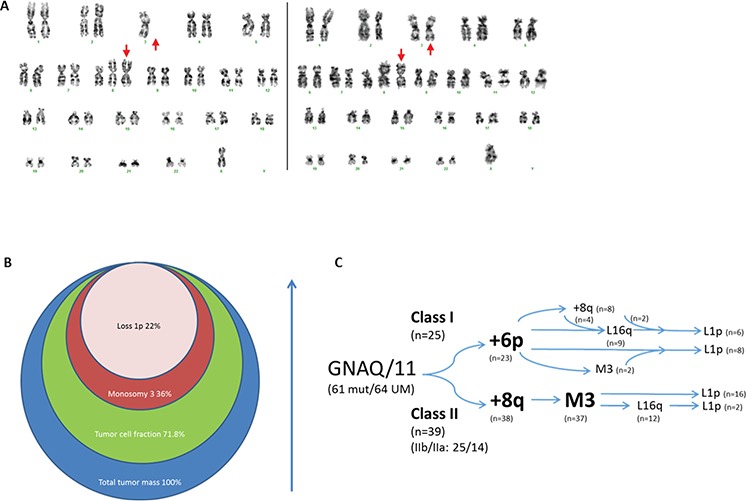
Chronology of genomic imbalances in UM development Karyograms of 06-023 revealed monosomy 3 heterogeneity while homogeneous isochromosome 8q indicated that it preceded monosomy 3 **A.** UM 20-042 displayed heterogeneity for monosomy 3 and loss of 1p, indicated by circles that represent the fractions of the tumor containing a specific chromosomal aberration **B.** Integration of tumor heterogeneity with gene-expression classes suggested distinct chronology in UM development in class I and class II **C.** Two UM in class I did not contain 6p gain but presented L1p and M3/8q+ genotypes respectively. The class II UM without gain of 8q did present monosomy 3.

Tumor heterogeneity was also determined with fractional abundance of the chromosomal imbalances. Moreover, quantification of tumor heterogeneity with dPCR provided information on the sequence of events. In UM 20-042 for example, 71.8% of the cells contained the GNAQ Q209P mutation while a chromosome 3 copy number of 1,64 indicated that 36% of the cells contained monosomy 3 and thereby revealed that monosomy 3 occurred after GNAQ mutation (Table 1). Because 8q copy number in UM varied widely we used karyotyping to order monosomy 3 and 8q gain instead (Figure 5A). Among 39 UM with monosomy 3 and 8q gain, three tumors (20-173, 06-015 and 06-023) displayed 8q gain in all tumor cells, but some cells contained two copies of chromosome 3. This indicates that 8q gain occurred before monosomy 3 in these tumors.

With dPCR calculated tumor fractions we furthermore deduced that loss of 16q seems to be more common as a quaternary event following monosomy 3. Ultimately, 1p loss occurs as the last event in this series of five recurrent aberrations (Figure 5B). For monosomy 3 and chromosome 16q loss, 14 tumors showed heterogeneity (ΔX > 0.2) and all showed a larger monosomy 3 tumor fraction. For the combination of loss of 16q and 1p, only six UM were informative and all showed a higher 16q tumor fraction compared to the 1p fraction. Temporal distribution of chromosomal aberrations can also be determined with the number of imbalances in a tumor and this was applied for 6p gain [24]. In class I, three UM presented only one imbalance and in all of these cases it concerned 6p gain. This indicated that in class I tumors, 6p gain most likely occurred first, following the GNAQ/11 mutation (Figure 5C).

## DISCUSSION

For UM excellent molecular markers exist to predict disease outcome [25]. Chromosomal imbalances and gene expression profiles accurately predict disease progression in UM but so far have not resulted in improved treatment. We set out to integrate expression and genomic profiles of UM to study the underlying mechanisms as this may facilitate a knowledge based treatment. By applying a rigorous data reduction to the gene expression analysis, we revealed three expression classes. These classes overlapped with classes that were identified by unsupervised cluster analysis but divided the UM with a bad prognosis into two groups that were designated class IIa and class IIb. The immune response and the translation machinery were the only two functions significantly enriched among the 237 genes that defined the three classes.

The five recurrent aberrations that were analyzed, were non-randomly distributed over the expression classes. In our tumor set, monosomy 3 and 6p gain clearly divided the tumors into class I and class II (a/b) (Figure 3). Based on the fact that monosomy 3 and 6p gain are present in different UM it is commonly assumed that these aberrations are mutually exclusive [26–28]. Quantification with digital PCR however showed some degree of 6p gain in combination with monosomy 3 and thereby indicates that the mutual exclusivity is not absolute. Though, our observations suggest that tumor clones presenting both monosomy 3 and 6p gain are unsuccessful and remain small. Class IIa and class IIb can be distinguished with 8q copy number. The number of chromosome 8q copies is highest in class IIb and this was related to a different underlying mechanism between classes. Class IIa more commonly yielded tumors with gain of a complete chromosome 8 while class IIb tumors almost exclusively displayed 8q copy gains due to isochromosome formation [23]. The formation of chromosome 8q isochromosomes is a phenomenon earlier described in UM [29–31].

Remarkably, most of the genes highly expressed in class IIb tumors are involved in immune regulation and located on chromosome 6 which is rarely gained in class II tumors. We therefore postulated that these differentially-expressed genes were not the result of differences in tumor cells, but expressed by stromal cells (e.g. fibroblasts and immune cells). This notion was sustained by our calculation of the tumor cell fraction in the total tumor mass. The tumor cell percentage was similar between class I and class IIa but was significantly lower in class IIb tumors suggesting a higher percentage of non-cancer cells in class IIb tumors. The role of the immune system in UM development has not yet been elucidated but correlations with survival and other prognostic markers have been made [32–34]. In UM, a bad prognosis is correlated with the presence of immune infiltrate [33] and an immune infiltrate may be what is causing the expression profile of class IIb (Table 2). Importantly, class IIa tumors that present a high tumor percentage, leaving few space for immune cell infiltrate, are also correlated with a bad prognosis. This implies that that the immune cells are not a prerequisite for the class IIa tumors to progress and metastasize.

The calculated tumor percentages of each chromosome loss furthermore allowed us to deduce the sequence of events in UM. Based on dPCR analysis we calculated which percentage of cells contained specific chromosomal losses (Figure 2). Because chromosomal gains could not be used to determine fractional abundance we used alternative approaches for 6p gain and 8q gain. In class I tumors 6p aberrations appear to occur first based on the fact that 22 out of 25 UM presented 6p gain in this class and because the tumors with only one chromosomal imbalance all presented 6p gain. Previous reports suggest that loss of chromosome 3 precedes the addition of 8q [35] but cytogenetic analysis of informative UM in our dataset supported the opposite. Karyotyping revealed monosomy 3 heterogeneity in tumors that are homogeneous for 8q gain and thereby suggested that chromosome 8q imbalance occurs prior to monosomy 3 in class II tumors. With regard to the sequence of chromosomal losses, we deduced that monosomy 3 occurs first, followed by loss of 16q and loss of 1p respectively

Surprisingly for such an early event, 8q copy number made molecular discrimination of the three gene-expression classes possible when we adjusted chromosome 8q copy number for tumor fraction. In a dosage-dependent manner, significant differences were revealed between the three expression classes. Previous studies showed that 8q copy number correlates with survival but no significant survival differences were revealed between class IIa and class IIb even though 8q copy number differed between these classes (Figure 4B). Both expression differences and genomic differences nevertheless suggest that class IIa and IIb represent different entities that may require different treatments.

In short, with integrated analysis of gene expression and genomic aberrations we were able to distinguish three molecular classes in UM. Based on an apparent immune infiltrate in class IIb we hypothesize that class II can progress either with or without immune involvement. Defining these classes gives us the opportunity to investigate the mechanism behind immune involvement in UM. Chromosome 8q is likely to be a candidate locus to search for targets since it is differentially changed in class IIa compared to class IIb. Dividing UM on a molecular level reveals tumor diversity and can lead to new possibilities for therapeutic intervention.

## MATERIALS AND METHODS

We used tumor material from 64 enucleated eyes of uveal melanoma patients that had been enucleated at the Leiden University Medical Center, Leiden, The Netherlands, between 1999 and 2008. Written informed consent was obtained for all patient samples. None of the tumors had prior treatment and we only used tumors with a follow-up time of at least 4 years. The maximum follow-up was 14 years. The average age at enucleation was 60.6 years (range 13 to 88); 33 patients were male and 31 female.

Tumor material was snap frozen using 2-methyl butane and RNA and DNA was isolated using the RNeasy mini kit and QIAmp DNA minikit, respectively, (both Qiagen, Valencia, USA) from 20 sections of 20 μm according to the manufacturer's guidelines.

### Gene expression profiling

Gene expression was determined using the Illumina HumanHT-12 v4 chip containing 47,000 probes across the whole genome that will be referred to as genes in the remainder of the text. Genes in the supervised cluster analysis with a log-fold change (LFC) larger than 1 or smaller than −1 and a *p*-value smaller than 0.05 were labelled “significantly differentially expressed”. For differences between subgroups i.e. I versus II, we corrected for differences between IIa and IIb classified as LFC smaller than −0.5 or greater than 0.5 and a *p*-value smaller than 0.05. These most differentially-expressed genes were annotated and biological processes were analyzed using the Database for Annotation, Visualization and Integrated Discovery (DAVID) [36, 37].

### SNP analysis

SNP microarray analysis was used to determine chromosomal aberrations. Two types of SNP microarray chips were used, the Affymetrix 250K_NSP-chip, with ~250,000 probes across the genome and the Affymetrix Cytoscan HD chip, with ~750,000. The first 28 samples were analyzed with the Affymetrix 250K_NSP chip, and the remaining 36 samples with the Affymetrix Cytoscan HD chip. Analysis of the Affymetrix 250K_NSP chips was performed with the ‘Genotyping Console’ to determine the copy number values and the ‘GCT Browser’ to visualize the data (both from Affymetrix, Santa Clara, USA). Affymetrix Cytoscan HD chips were analyzed with ‘ChAS’. Different loci per chromosome were evaluated to adjust for partial gains or deletions. ~200 probes per gene locus were averaged to determine eventual copy number.

### dPCR

#### Copy number analysis

The copy number of chromosome 1p, 3, 6p, 8q, and 16q was determined using probes for *CDC42*, *PPARG*, *NEDD9, PTK2*, and *NFAT5* respectively. For calculation of normalized copy numbers, out of 3 control probes located on chromosome 5, 7, and 14 (*TERT, VOPP1 and TTC5)*,*TERT* (situated at chromosome 5), was selected based on stability in this tumor set. In the two cases with chromosome 5 gain we used *TTC5* as reference to calculate the copy numbers. Thresholds for copy number analysis were: loss, <1.9: normal, 1.9–2.1: gain, >2.1− <3.1: amplification, >3.1 (method described by Versluis *et al*. [23]). In short, 50–60 ng of DNA of each sample was used in a 20 ul reaction volume. Reaction mixture consisted of 2x droplet PCR supermix (Bio-Rad Laboratories, Inc., Hercules, USA), 20x target probe (FAM), 20x reference probe (HEX). Sequence context is provided in Supplementary Table S1. Droplets were generated using a QX100 droplet generator and after the PCR, the plate was loaded into the QX100 droplet reader (both Bio-Rad Laboratories, Inc.). The following end point PCR protocol using a T100 thermal cycler was used: 95°C, 10 min; (94°C, 30sec; 60°C, 1min) 40x; 98°C, 10 min; 4°C, till end. Digital PCR (dPCR) software (QuantaSoft) reads the positive and negative droplets in each sample and plots the fluorescence droplet by droplet. Fractional abundance was calculated as $FA=\frac{mutant\;amplicons}{mutant\;amplico\;+\;wildtype\;amplicons}$. The positive droplets represent the concentration of the target allele in the sample. Corrections made for the fraction of tumor cells containing chromosomal aberrations (Z) based on FA were performed as follows: X represents the dPCR value for copy number of chromosome *n*, and Y represents the tumor fraction of the associated tumor which is calculated by multiplying the FA by 2.

Heterogeneity of imbalances was determined by subtracting copy numbers and a difference of 0.2 copy number was used as threshold (ΔX > 0.2).

$$Z=\frac{X-\left(2\left(1-\left(\frac{Y}{100}\right)\right)\right)}{\frac{Y}{100}}$$

#### GNAQ/11 mutation detection

GNAQ and GNA11 mutations were detected using hydrolysis probes in a multiplex dPCR. 10 ng of sample DNA was used in a 20 ul reaction volume. The reaction mixture consisted of 2x droplet PCR supermix (Bio-Rad Laboratories, Inc.), 20x target probe (FAM), and 20x wildtype probe (HEX). Proprietary probes and primers (Bio-Rad Laboratories, Inc.) were used and the sequence context is provided in Supplementary Table S1. Droplet generation, thermal cycling and reads were similar to the method described in the previous section. PCR to end point protocol used: 95°C, 10 min; (94°C, 30 sec; 55°C, 1 min) 40x; 98°C, 10 min; 4°C, till end.

### Karyotyping

Following enucleation, a small part of each tumor was sent out for cell culture. Following mechanical dissection of the tumor biopsy, cells were washed and placed into one flask with RPMI 1640 (15% fetal bovine serum [Invitrogen, Breda, The Netherlands]) medium and another flask with Amniochrome II (Cambrix Bio Science, Verviers, Belgium). The flasks were cultured at 37°C with 5% CO_2_ for up to 4 weeks and harvested when at least 75% of the surface was covered with cells (after a mean of 18 days; SD, 9.4 days). When cell culturing was successful, conventional karyotyping was performed, to determine the presence of chromosomal changes. Two independent observers assessed all evaluations and scores, each without knowledge of the results obtained by the other investigator, to ensure accuracy of quantification of the slides. In case of a difference, consensus was reached during a simultaneous session. Cytogenetic analysis was performed on GTG-banded (G-banding with trypsin and Giemsa) metaphases. In the case of a normal karyotype, at least 20 metaphases were analyzed. When an abnormal clone was detected in the first ten karyotyped cells, no further analysis was performed; when three cells with loss of 1 copy of chromosome 3 were observed, monosomy 3 was identified.

### Statistical analysis

For gene expression analysis, the statistical programming language R was used (R: A Language and Environment for Statistical Computing, R Core Team, R foundation for Statistical Computing, Vienna, Austria, 2014). Since data have been obtained in two batches, a batch effect correction was applied. The R packages used were: ‘ber’ for batch correction and ‘lumi’ for unsupervised clustering. To compare survival between UM patients in different classes Kaplan-Meier functions were plotted. Survival analysis was performed using the log-rank test. The Pearson's correlation test was used to assess the correlation between the fraction of tumor cells containing GNAQ/11 and the fraction of tumor cells containing chromosome 3 aberrations. The Chi square test was used to test frequency differences in chromosomal aberrations in different subgroups. Likelihood ratios were used for cases in which the Chi square assumption was violated. *α* = 0.05 was used as threshold for significance in all tests. For statistical analysis SPSS V.20.0.1 (IBM SPSS statistics, IBM corporation, Armonk, New York, USA) was used.

## SUPPLEMENTARY FIGURES AND TABLES


