# Supplementary material for: Heterogeneity revealed by integrated genomic analysis uncovers a molecular switch in malignant uveal melanoma
Source: Oncotarget. 2015 Oct 8;6(35):37824–35. doi: 10.18632/oncotarget.5637 (PMC4741968; doi:10.18632/oncotarget.5637)
Supplement: Supplementary file 1 [file oncotarget-06-37824-s001.pdf]

SUPPLEMENTARY FIGURES AND TABLES

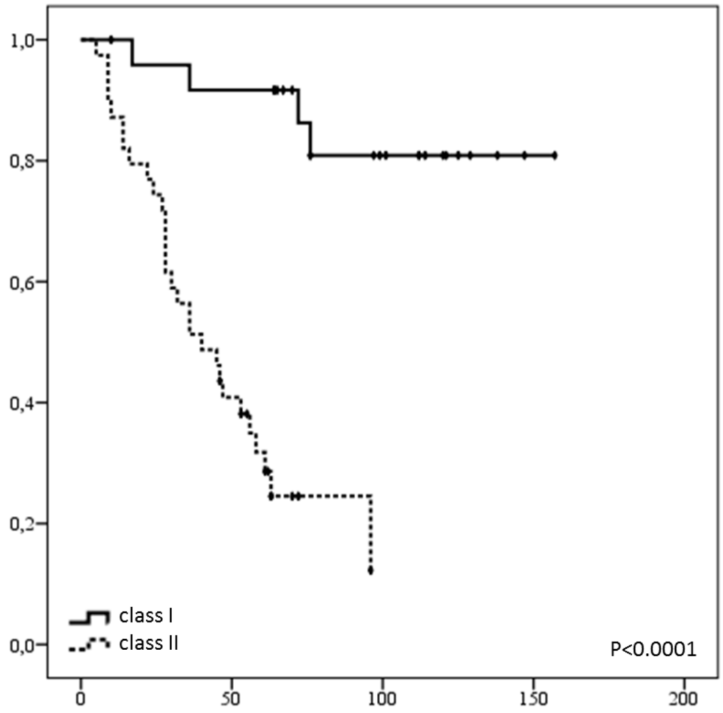

Supplementary Figure S1: Kaplan-Meier analysis revealed a clear difference in survival between class I and class II.

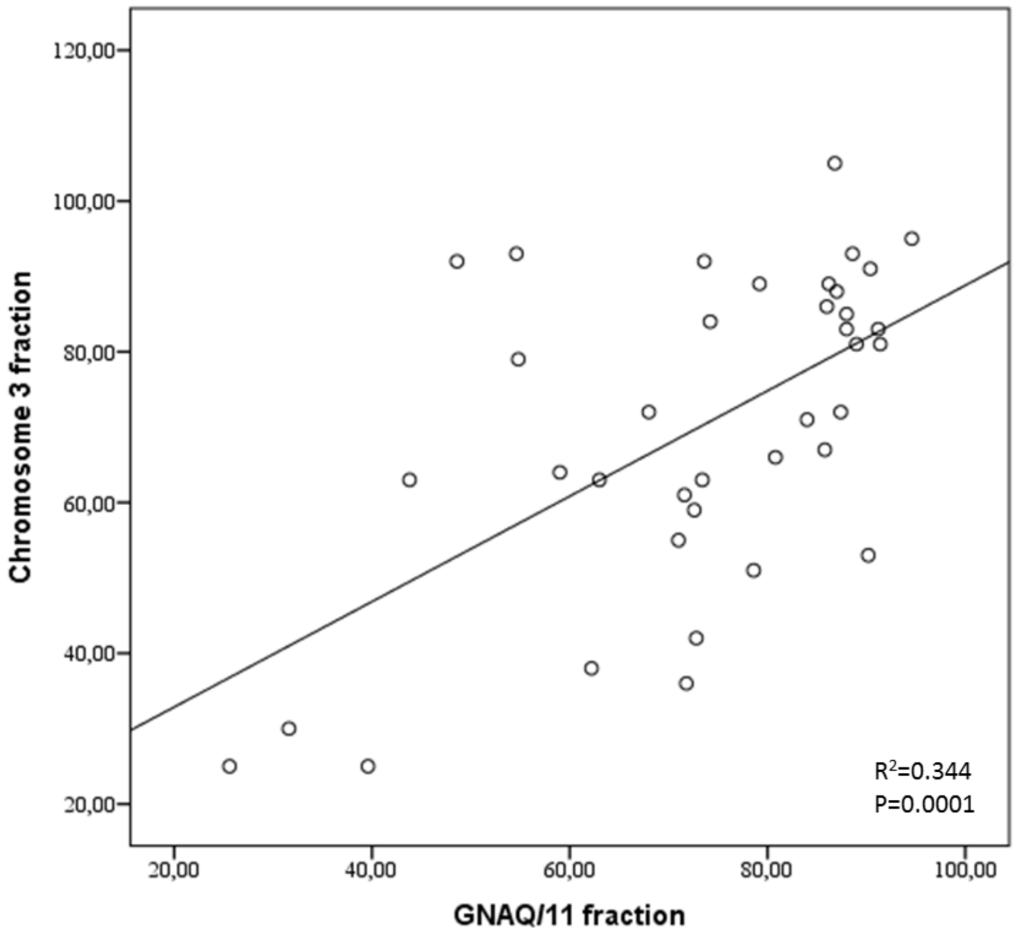

Supplementary Figure S2: Correlation between fraction of tumor cells containing a GNAQ/11 mutation and fraction of tumor cells containing monosomy 3. A significant correlation indicates that monosomy 3 fraction can be used to determine tumor cell fraction in tumors which lack a GNAQ/11 mutation.

Supplementary Table S1: dPCR sequences context

Assay	MiQE Context Sequence
GNAQ Q209	AGTGTATCCATTTTCTTCTCTCTGACCTTTGGCCCCCTACATCGACCATTCTGCAAGGTTAA CAATACTCATATTAATAACATATAAAGTAAAACTAAAAAGTCAACATAAATATAGCACTAC
GNA11 Q209	CTTTCAGGATGGTGGATGTGGGGGGCCAGCGGTCGGAGCGGAGGAAGTGGATCCACTGCTTT GAGAACGTGACATCCATCATGTTTCTCGTCGCCCTCAGCGAATACGACCAAGTCCTGGTGG
TERT	CACCCCTTGGTGGCGGCTCACCTGTACGCCTGCAGCAGGAGGATCTTGTAGATGTTGGTGCA CACCGTCTGGAGGCTGTTCACCTAGAGTCGCCAAGAAAGAGTGAGAAACGGTAGAAACCTC
PPARG	TCTCCACCTTATTATTCTGAGAAGACTCAGCTCTACAATAAGCCTCATGAAGAGCCTTCCAAC TCCCTCATGGCAATTGAATGTCGTGTCTGTGGAGATAAAGCTTCTGGATTTCACTATGGA
PTK2	CAACCAGATGGTCATTCAAAAAAGTTGGAGCTGTAAGTGCTGGCGACTGAGGACACAGGGTT AATTCCTCGCTGCTGGTGGAAGGCTAGAGAACATCTTCAAAAGAGGGTAGCAAGACGTGCT
CDC42	TTCACACACTTAGCTTTTTTTTGTGATGAGAACATTTAAAATATACTCACTTAGCAATTTTCAA GTGTGTAATACCGTATTATTAACTATAGTCACCATGCTGTACGGTAGCTCTCCAGAACG
NEDD9	GCTCCCCAGGCTGGAGTACAGTGGCACAATCTCAGCTTACAGCAACCTCTGCCTCCCGGGTT CAAGTGATTTTCCTGCCTCAGCCTCCTGAGTAGTTGAGATTACAGGTGCACGCCACCATGC
NFAT5	TGGAAGCCATGAGAGAGAATATGATTTCAGGAAAACTTGGGCACAGAGAATTTGGCCTATCTC AGTATGACAAACTAATAGCTGTTGTGATTCATTTAGGGACTGTTGTTAAATATTTGATTA

Supplementary Table S2: Gene expression of the top differentially expressed genes in the three classes

Probe	Map	Gene Symbol	LogFC.1 vs 2	Adj. *p*-value 1 vs 2	LogFC.2A vs 2B	Adj. *p*-value 2A vs 2B	LogFC.1 vs 2A	Adj. *p*-value 1 vs 2A
ILMN_1735764	2q36.3-q37.1	HTR2B	−3.773908392	3.32E-14	−0.934860257	0.065334695	−3.199143842	1.15E-10
ILMN_1803945	6p21.3	HCP5	−2.66663481	2.95E-10	−1.526639411	0.00433641	−1.666679192	5.21E-05
ILMN_1778401	6p21.3	HLA-B	−2.38907834	2.45E-08	−2.081580926	0.000862178	−1.03733205	0.018977868
ILMN_1691641	Xq13.1	CITED1	−2.287132646	5.18E-10	−0.171011652	0.585474286	−2.167603733	6.73E-07
ILMN_2337655	14q32.31	WARS	−2.266810219	4.91E-09	−1.805074178	0.000158268	−1.194667337	0.000551673
ILMN_1727271	14q32.31	WARS	−2.143112717	2.17E-08	−1.780051251	0.000301266	−1.064401571	0.00231439
ILMN_1770940	16q22.1	CDH1	−2.076563054	2.36E-07	0.867038878	0.044872618	−2.652152735	7.75E-08
ILMN_2058782	14q32	IFI27	−2.036064217	0.000495533	−2.952315446	0.000341596	−0.053944659	0.964550758
ILMN_1751079	6p21.3	TAP1	−1.999461061	3.00E-07	−2.17396159	1.55E-05	−0.713550773	0.04172581
ILMN_2329735	1q21	ECM1	−1.996423647	4.22E-09	−0.862975474	0.097090624	−1.432839125	7.84E-05
ILMN_2352097	16q13	GPR56	−1.978357632	1.40E-08	0.06638202	0.880409419	−2.089506775	5.00E-07
ILMN_1720282	16q22.1	NQO1	−1.958702811	7.01E-12	−0.312744968	0.456598454	−1.744600311	3.40E-08
ILMN_1724533	8q21.11	LY96	−1.943487835	5.42E-05	−1.61796231	0.026150643	−0.727712908	0.303492543
ILMN_2384122	16q13	GPR56	−1.938534966	1.06E-08	0.080961406	0.84253637	−2.057689005	3.03E-07
ILMN_2188862	19p13.11	GDF15	−1.920898369	5.02E-06	−0.689602297	0.218668928	−1.386779452	0.009212452
ILMN_1657111	14q32.33	AHNAK2	−1.904421005	2.83E-07	0.19839201	0.79063382	−1.967675258	4.25E-05
ILMN_1691364	2q32.2	STAT1	−1.901311636	3.87E-06	−2.22685769	0.000156814	−0.519941698	0.268249908
ILMN_2186806	6p21.3	HLA-A	−1.856722458	1.24E-06	−1.842512556	0.000548884	−0.680184582	0.125312752

(*Continued*)

Probe	Map	Gene Symbol	LogFC.1 vs 2	Adj. *p*-value 1 vs 2	LogFC.2A vs 2B	Adj. *p*-value 2A vs 2B	LogFC.1 vs 2A	Adj. *p*-value 1 vs 2A
ILMN_1758128	17q25	CYGB	−1.849044334	2.74E-08	−0.134359598	0.839360217	−1.877894072	2.25E-06
ILMN_2130441	6p21.3	HLA-H	−1.837938204	2.68E-07	−1.827910037	5.54E-05	−0.661482354	0.091379956
ILMN_2049536	17p11.2	TRPV2	−1.726229181	3.32E-08	−0.226198045	0.745153761	−1.49996204	9.93E-05
ILMN_2054019	1p36.33	ISG15	−1.710533103	1.79E-05	−2.218751047	5.35E-05	−0.342756166	0.480783875
ILMN_1784602	6p21.2	CDKN1A	−1.637679023	6.72E-08	−0.382698788	0.549429049	−1.305559946	0.001062968
ILMN_3243156	14q32.33	AHNAK2	−1.631469761	4.57E-07	0.245578987	0.676536857	−1.717288078	4.15E-05
ILMN_1723978	22q13.1	LGALS1	−1.607783419	5.87E-11	−0.597722926	0.040683991	−1.13374455	0.000225399
ILMN_1801246	11p15.5	IFITM1	−1.603895174	7.09E-05	−2.172972454	5.54E-05	−0.2908723	0.609435438
ILMN_1660691	18p11.3	RAB31	−1.582171721	2.07E-11	−0.163211795	0.677888809	−1.401483179	5.75E-07
ILMN_2108735	20q13.3	EEF1A2	−1.581954568	0.003061057	−1.256779356	0.143336167	−0.812554953	0.294247268
ILMN_1676563	10q26.3	HTRA1	−1.571761855	1.06E-08	−0.281216032	0.578241003	−1.25839444	0.00029402
ILMN_1666078	6p21.3	HLA-H	−1.56693276	0.000220765	−1.492089818	0.011779147	−0.804267994	0.118692468
ILMN_1694268	2q37.3	HES6	−1.551730975	3.51E-10	0.010207587	0.987060183	−1.389314129	1.94E-05
ILMN_2379644	5q32	CD74	−1.522558292	0.000208917	−1.456207652	0.05047505	−0.575269248	0.352677712
ILMN_1704154	13q12.11-q12.3	TNFRSF19	−1.50147218	1.67E-07	−0.17432277	0.810775999	−1.3172177	0.000305706
ILMN_2060086	2q33	ADAM23	−1.494049148	1.24E-05	0.892425379	0.099186067	−2.080566429	1.36E-06
ILMN_2366330	11q13.1	FERMT3	−1.482467027	1.63E-08	−0.155069949	0.726916522	−1.323238034	4.78E-05
ILMN_1725427	15q21-q22.2	B2M	−1.481512737	3.54E-07	−1.27677156	0.002141	−0.667950409	0.035732787
ILMN_1679208	7q22.1	LHFPL3-AS1	−1.477219872	4.57E-07	−0.498307113	0.329942711	−1.000683167	0.005230774
ILMN_1736567	5q32	CD74	−1.475482233	0.000347899	−1.451771578	0.054889055	−0.528228843	0.412408716
ILMN_2148459	15q21-q22.2	B2M	−1.474818568	1.09E-08	−1.128232399	0.000518798	−0.733307622	0.005499572
ILMN_2390299	6p21.3	PSMB8	−1.462913166	3.24E-07	−1.489399362	5.54E-05	−0.464486376	0.127891696
ILMN_1653494	1q21	S100A1	−1.456593391	0.000100276	0.108023535	0.895535539	−1.615577519	0.000696915
ILMN_1793476	11p15.4	PRKCDBP	−1.419121924	5.40E-07	−0.412504148	0.449002125	−1.026111852	0.00681702
ILMN_1747195	6p21.3	PSMB8	−1.406736629	2.33E-07	−1.361023129	0.000142967	−0.484940728	0.090678383
ILMN_1796409	1p36.12	C1QB	−1.40534744	0.000681717	−1.495300525	0.026994241	−0.448802397	0.484071008
ILMN_1659913	15q26	ISG20	−1.403091805	3.91E-05	−1.549345257	0.00060989	−0.306617422	0.478974807
ILMN_1725193	2q33-q34	IGFBP2	−1.399708363	3.09E-08	−0.12908906	0.843863729	−1.162161451	0.000466155
ILMN_1782050	8p11.2-p11.1	CEBPD	−1.395006423	4.39E-07	−0.043338611	0.952858182	−1.334197316	0.000210616
ILMN_2157441	6p21.3	HLA-DRA	−1.393274828	0.001410494	−1.738923346	0.014976541	−0.282501616	0.717775178
ILMN_1769779	8q24.3	PTP4A3	−1.39004584	1.31E-07	−0.484935905	0.252641849	−1.100111001	0.000280978
ILMN_1762861	6p21.3	HLA-F	−1.351637038	1.98E-05	−1.385331383	0.003045645	−0.350139811	0.41508905
ILMN_1708375	5q31.1	IRF1	−1.343649903	3.26E-05	−1.432841362	0.001588993	−0.381627053	0.34522034
ILMN_1810844	7q36.1	RARRES2	−1.335158883	0.000222874	−0.062936549	0.944543429	−1.165391772	0.022203596
ILMN_1765258	6p21.3	HLA-E	−1.311146182	7.66E-06	−1.611888624	0.000158268	−0.315797125	0.409305273
ILMN_1772218	6p21.3	HLA-DPA1	−1.310768829	0.002836507	−1.453661429	0.061661454	−0.357607473	0.640730802
ILMN_1729691	17q24.2	SLC16A6	−1.299771314	3.62E-06	−0.090602799	0.913423939	−1.231147812	0.000626107
ILMN_1713636	1q21	S100A6	−1.294152227	2.53E-07	−0.638913429	0.120437702	−0.895606445	0.00325878
ILMN_1702487	6q23	SGK1	−1.291490458	0.001490199	−0.294082571	0.718613535	−0.928152744	0.129251968
ILMN_2109708	22q13.33	TYMP	−1.2878876	2.21E-05	−1.512205093	0.000283042	−0.327015501	0.446610528

(*Continued*)

Probe	Map	Gene Symbol	LogFC.1 vs 2	Adj. *p*-value 1 vs 2	LogFC.2A vs 2B	Adj. *p*-value 2A vs 2B	LogFC.1 vs 2A	Adj. *p*-value 1 vs 2A
ILMN_1795930	5p13.1	PTGER4	−1.286717865	0.000970005	−0.76748395	0.18230754	−0.606010589	0.336703663
ILMN_2098126	17q11.2-q12	CCL5	−1.284655576	0.002322207	−1.86024864	0.008616187	−0.060827329	0.948879493
ILMN_2358069	7p22	MAD1L1	−1.274162082	1.04E-08	0.081873277	0.89705128	−1.250656388	1.11E-05
ILMN_2359710	8q24.3	PTP4A3	−1.273250293	2.03E-07	−0.419965606	0.335068999	−1.002959441	0.000564678
ILMN_1696187	14q21-q22	PYGL	−1.254681852	0.001771987	0.453479289	0.60410177	−1.648594315	0.001355887
ILMN_1730084	22q11.21	COMT	−1.253157567	5.73E-12	0.298837526	0.245428188	−1.398281728	4.04E-10
ILMN_1806733	21q22.3	COL18A1	−1.248432723	4.63E-09	−0.13563169	0.807207586	−1.09196204	4.15E-05
ILMN_1785902	1p36.11	C1QC	−1.242953802	0.000337762	−1.292361987	0.017742391	−0.423036425	0.392003425
ILMN_1761733	6p21.3	HLA-DMB	−1.23681126	0.001074151	−1.409926005	0.016646807	−0.208680975	0.783227001
ILMN_1739241	15q15.1	CHAC1	−1.233630321	4.86E-11	−0.097759854	0.807164842	−1.153413343	4.53E-07
ILMN_1757387	4p14	UCHL1	−1.223234587	0.000958245	−0.627741177	0.217766395	−0.485457064	0.453521545
ILMN_1669523	14q24.3	FOS	−1.22144887	0.000151106	−0.264449692	0.721002588	−1.057807158	0.012502918
ILMN_1682928	7p15.1	CPVL	−1.213052895	2.24E-08	−0.199378838	0.415111615	−1.158055933	2.09E-06
ILMN_2062468	4q12	IGFBP7	−1.212651879	3.52E-05	0.217779953	0.677086483	−1.451266092	7.88E-05
ILMN_1786041	Xp22.2	ASB9	−1.209130965	5.63E-08	0.282118814	0.488074948	−1.441938823	1.10E-07
ILMN_1779171	17p13.3	SGSM2	−1.20596083	5.37E-07	0.432776886	0.122474351	−1.583182598	5.85E-08
ILMN_1662427	8q24.3	PTP4A3	−1.205823574	3.09E-07	−0.415432091	0.296312507	−0.941502922	0.000743735
ILMN_1671054	6p21.3	HLA-A	−1.205252456	4.13E-07	−1.033963813	0.001992219	−0.434241785	0.150599387
ILMN_1662358	21q22.3	MX1	−1.203899161	0.000440498	−1.673818424	0.002630017	−0.189552499	0.769106644
ILMN_1687384	1p35	IFI6	−1.198258771	0.000442643	−1.847146326	0.000301266	−0.04565734	0.953193267
ILMN_1663866	5q31	TGFBI	−1.189847867	3.17E-06	−0.508328367	0.290550661	−0.869434873	0.005514915
ILMN_1656194	17q25.3	TSPAN10	−1.188484584	4.56E-06	0.150583817	0.773596208	−1.310845191	0.000128899
ILMN_1777325	2q32.2	STAT1	−1.18802805	0.000202326	−1.895540123	5.54E-05	0.003009297	0.996141387
ILMN_1676099	4p16.3	SPON2	−1.177578697	3.55E-09	−0.177833628	0.686698836	−0.933368764	0.00034736
ILMN_2229379	4q11-q12	KIT	−1.176612669	0.000936995	0.735803564	0.176446393	−1.815791705	0.000101932
ILMN_1789733	19q13.12	CLIP3	−1.171904708	1.50E-06	0.161867421	0.730052968	−1.159783617	0.000639028
ILMN_1689655	6p21.3	HLA-DRA	−1.167043216	0.007892476	−1.613776991	0.027715304	−0.135281356	0.878552409
ILMN_1710740	6p21.3	C2	−1.164851011	6.17E-10	−0.424882045	0.098916056	−0.871550238	2.21E-05
ILMN_1801766	4q25	CCDC109B	−1.163072045	6.52E-08	−0.504777282	0.109892945	−0.701162306	0.00977496
ILMN_1790160	4q11-q12	KIT	−1.161790871	0.002217089	0.963724842	0.104580216	−1.901990254	0.000231025
ILMN_1656670	6p21.3	HLA-G	−1.155427467	7.79E-06	−1.153720079	0.000555697	−0.403899161	0.196509477
ILMN_2366212	17q23	CD79B	−1.152695659	3.99E-05	−0.252558905	0.636624936	−1.083677951	0.002516523
ILMN_2400759	7p15.1	CPVL	−1.152219072	1.24E-07	−0.219539557	0.405082416	−1.107281891	1.03E-05
ILMN_1805750	11p15.5	IFITM3	−1.146116484	3.97E-08	−0.665509972	0.015898115	−0.727714558	0.002113514
ILMN_1688780	1q21	S100A4	−1.145891451	0.000928153	−0.8091866	0.132261453	−0.569235362	0.264284082
ILMN_2203950	6p21.3	HLA-A	−1.145672159	3.38E-09	−0.779088685	0.000856288	−0.641404042	0.002494604
ILMN_1690105	2q32.2	STAT1	−1.143448518	0.000309827	−1.36174288	0.002120612	−0.180381399	0.709423443
ILMN_1771800	17q22-q23.2	PRKCA	−1.124829082	4.51E-05	−0.3701347	0.503222467	−0.903227909	0.011468085
ILMN_2067656	12p13	CCND2	−1.120127725	0.004083695	0.475659118	0.420139451	−1.418441381	0.006122274
ILMN_1774077	1p22.2	GBP2	−1.117392306	0.004043561	−1.715583799	0.00433641	0.004970278	0.995396575

(*Continued*)

Probe	Map	Gene Symbol	LogFC.1 vs 2	Adj. *p*-value 1 vs 2	LogFC.2A vs 2B	Adj. *p*-value 2A vs 2B	LogFC.1 vs 2A	Adj. *p*-value 1 vs 2A
ILMN_1815115	8q24.3	CYC1	−1.111580082	4.63E-08	−0.554010798	0.108902369	−0.76621322	0.000989067
ILMN_1722820	22q13.1	KDELR3	−1.110198917	4.14E-08	−0.370423955	0.18281206	−0.905728416	5.90E-05
ILMN_1808405	6p21.3	HLA-DQA1	−1.107343321	0.015565072	−1.444103747	0.054603468	−0.095545379	0.922552961
ILMN_1802027	4q28.3	MGST2	−1.101678462	1.06E-08	−0.361167552	0.12459274	−0.929274922	1.07E-05
ILMN_1695311	6p21.3	HLA-DMA	−1.094127489	0.000890934	−1.352001983	0.009311445	−0.225074599	0.709598127
ILMN_1743103	10q24.33	SH3PXD2A	−1.08906427	3.38E-06	0.607414723	0.137438736	−1.400508743	3.78E-06
ILMN_2334296	11q13	IL18BP	−1.082418546	0.00016257	−1.191449854	0.002971623	−0.201727242	0.679569868
ILMN_2066066	6p21.3	HLA-DRB6	−1.081050989	0.004337548	−1.420112933	0.019127522	−0.106183998	0.892864946
ILMN_1708778	9q34.1	ASS1	−1.07728373	2.91E-05	−0.364980639	0.368764337	−0.637488249	0.095475721
ILMN_1803788	14q22.3	LGALS3	−1.070982922	1.04E-05	−0.822964131	0.011779147	−0.480980901	0.155314416
ILMN_1673352	11p15.5	IFITM2	−1.070116856	1.12E-08	−0.698340489	0.004154219	−0.653207814	0.00146188
ILMN_3245057	8q24.1-q24.2	ASAP1	−1.068044193	5.13E-07	0.251282236	0.565761753	−1.172244835	2.41E-05
ILMN_3305938	6q23	SGK1	−1.064550475	0.007636294	−0.290727373	0.710479608	−0.690151371	0.274836645
ILMN_1805737	10p15.3-p15.2	PFKP	−1.064253058	1.13E-07	−0.281986458	0.227127929	−0.842631468	0.00049186
ILMN_1684306	1q21	S100A4	−1.055266309	0.001034069	−0.769577479	0.103832364	−0.507951039	0.285227807
ILMN_1686664	16q13	MT2A	−1.050536453	0.00018572	−0.813068892	0.060627835	−0.496255256	0.207895987
ILMN_1745034	12q13	SLC11A2	−1.045247158	3.87E-08	−0.100349454	0.822909956	−0.953501469	8.71E-05
ILMN_1787345	12q13.12	FKBP11	−1.044617402	0.000305274	−0.615553014	0.254352932	−0.691177225	0.094597515
ILMN_1701613	11q23	RARRES3	−1.04129153	0.028213669	−2.117220714	0.001197527	0.359788873	0.590200319
ILMN_1771652	22q13.1	BAIAP2L2	−1.037245324	9.58E-07	0.08336743	0.83299303	−1.186731927	3.83E-06
ILMN_1743836	17q25.1	MXRA7	−1.035744309	9.22E-11	0.122853077	0.718989023	−1.066988757	6.25E-08
ILMN_1656920	14q32.33	CRIP1	−1.035138968	6.35E-07	−0.571091783	0.091294504	−0.610058978	0.018735274
ILMN_1766657	9q34.1	STOM	−1.032720922	8.73E-07	−0.686220443	0.03717254	−0.5476821	0.043531095
ILMN_1778977	19q13.1	TYROBP	−1.026783881	0.004074888	−1.285362278	0.012378171	−0.056575673	0.945469006
ILMN_3229324	6q23	SGK1	−1.017792894	0.015168731	−0.271510437	0.748202438	−0.63921103	0.349488079
ILMN_2218208	4q22.1	SPARCL1	−1.016969117	0.026934898	0.840276126	0.405034431	−1.532358514	0.010204208
ILMN_1744118	9q33.1	ASTN2	−1.013255179	1.37E-05	−0.038780848	0.957168549	−0.983087258	0.001245753
ILMN_1736982	6p24.1	PHACTR1	−1.010594848	0.047662066	−0.495126318	0.535259965	−0.58128186	0.494308796
ILMN_1676893	2p23.3	ADCY3	−1.010294916	2.34E-08	−0.196587678	0.521295588	−0.7775104	0.001426401
ILMN_1725791	10p14-p13	PTPLA	−1.010177268	8.73E-07	−0.19282835	0.678530722	−0.874405961	0.000700149
ILMN_1773352	17q11.2-q12	CCL5	−1.010089785	0.002187047	−1.247163489	0.022681411	−0.082984494	0.903532691
ILMN_2376108	6p21.3	PSMB9	−1.00615672	0.000970071	−1.245073429	0.003045645	−0.001525824	0.998188548
ILMN_1723480	19p13.1	BST2	−1.00536494	0.002547297	−1.616081005	0.000486479	0.067795293	0.912632742
ILMN_1732923	1q42.2	SIPA1L2	−1.001007275	7.61E-09	0.248899188	0.354864633	−1.221156841	1.98E-08
ILMN_2227817	6p12.1	GSTA3	1.000389382	0.007978159	0.32219132	0.283738698	0.885142665	0.091525165
ILMN_3210171	1q32.3	RPL21P28	1.009169659	2.01E-05	−0.043780051	0.956450371	0.925899886	0.005180224
ILMN_2230025	4q35	PDLIM3	1.012908713	0.000827357	0.118682307	0.832217916	0.969901392	0.032247858
ILMN_1729033	4p13	RPL9	1.01384583	3.88E-08	0.106650368	0.836323157	0.912498289	0.000670585
ILMN_3225591	3p22-p21.2	RPL14	1.020358629	1.14E-12	0.254346968	0.199791869	0.84168109	3.68E-08
ILMN_1691611	19p13.2	HNRNPA1P10	1.025336757	2.74E-08	0.409599232	0.209889412	0.647860311	0.011950508

(*Continued*)

Probe	Map	Gene Symbol	LogFC.1 vs 2	Adj. *p*-value 1 vs 2	LogFC.2A vs 2B	Adj. *p*-value 2A vs 2B	LogFC.1 vs 2A	Adj. *p*-value 1 vs 2A
ILMN_3304898	6p21.33	TUBB	1.025842056	5.31E-08	0.256964706	0.344464336	0.797557368	0.00151551
ILMN_1699562	8q11.21	EFCAB1	1.026617421	0.000220765	0.063224105	0.480255267	1.020701496	0.004029976
ILMN_1774823	4q25	RPL34	1.03941488	1.56E-08	0.093231041	0.789770273	0.964168005	0.000221881
ILMN_3278506	7p15.3	RPS2P32	1.040920931	1.21E-06	0.177597564	0.747809982	0.894309529	0.004914792
ILMN_2325506	20q13.13	BCAS4	1.043939436	1.82E-05	−0.183413303	0.608029491	1.273257967	2.78E-05
ILMN_1740927	6p25.1	LYRM4	1.046906568	2.07E-11	0.242269164	0.109745518	0.88699802	1.47E-07
ILMN_2389273	3q28	FXR1	1.047553084	1.04E-10	0.215593781	0.102435441	0.88530183	3.31E-06
ILMN_1766165	4q21	SNCA	1.051796428	5.47E-06	0.28018814	0.302800751	0.864838221	0.003237044
ILMN_1654609	5q22.2	EPB41L4A-AS1	1.054896923	1.60E-08	0.538245025	0.083463239	0.649568228	0.008895082
ILMN_1797154	7q22.1	AZGP1	1.057343839	2.84E-08	0.178031986	0.120437702	0.907332771	4.34E-05
ILMN_1808059	20q13.13	BCAS4	1.058574197	5.55E-06	−0.137182546	0.68512161	1.263332358	1.05E-05
ILMN_1741096	8p23.1-p22	FDFT1	1.066405633	8.09E-07	0.525631817	0.170094483	0.68629575	0.012896564
ILMN_3293685	16p13.3	RPS2	1.072483613	4.54E-06	0.093858595	0.905768961	0.923115148	0.005843499
ILMN_1679093	19q13.42	ZNF581	1.072600242	1.67E-10	0.197124122	0.335375027	0.888933663	2.15E-05
ILMN_1665824	6p24.3	LINC00518	1.074925305	4.20E-08	0.149632708	0.366535017	0.771126237	0.005119079
ILMN_3302177	3p22-p21.2	RPL14	1.077184959	3.36E-11	0.241119376	0.368764337	0.930657812	8.95E-07
ILMN_1680344	18p11.31	MYOM1	1.078054364	0.000271898	−0.087273802	0.664111921	1.201942327	0.001839539
ILMN_1808041	6p21.31	RPL10A	1.082382125	3.29E-06	0.195958938	0.747678818	0.89521463	0.004843674
ILMN_1654946	19q13.43	ZSCAN18	1.082955746	4.22E-09	0.279033998	0.385037485	0.884732152	4.91E-05
ILMN_2144088	8p23.1-p22	FDFT1	1.086032559	2.37E-06	0.608412996	0.149593528	0.704375182	0.01383804
ILMN_1664292	19q13.42	ZNF415	1.088629313	2.40E-14	−0.060568205	0.450118757	1.173901165	1.41E-14
ILMN_2351638	Xq22.1-q22.3	BEX4	1.08871681	3.42E-10	0.266483391	0.258920998	0.870348102	2.78E-05
ILMN_2138765	9p22.1	PLIN2	1.0950394	3.00E-08	−0.341854853	0.24190242	1.366876809	1.98E-08
ILMN_1679640	3q28	FXR1	1.0988054	2.31E-12	0.266476463	0.080969607	0.944250501	3.48E-09
ILMN_1810533	12q21.3	SLC6A15	1.100594841	1.90E-08	0.335476464	0.213403899	0.826079503	0.000784998
ILMN_1766446	6p21.3	C6orf48	1.100937012	3.25E-09	0.226332046	0.340211668	0.927314751	2.67E-05
ILMN_1660439	22q13	RPL3	1.106517102	1.76E-07	0.40250656	0.263954692	0.763851676	0.006599111
ILMN_3244583	5q22.2	EPB41L4A-AS1	1.108311628	6.78E-09	0.545709662	0.055987692	0.692940462	0.004698967
ILMN_1694106	3p22.3	GPD1L	1.110684568	1.10E-09	0.392620984	0.047867867	0.823978019	4.25E-05
ILMN_3225784	9q21.13	RPSAP9	1.114453354	4.02E-10	0.209152773	0.435478729	1.028823175	1.61E-07
ILMN_2404850	3p22-p21.2	RPL14	1.128421289	0.000912076	0.477220804	0.382725969	0.648983261	0.207973525
ILMN_3241051	3p25-p24	RPL32	1.144777125	1.68E-11	0.377499734	0.081451755	0.896340608	6.54E-07
ILMN_1762747	3p24.2	RPL15	1.147236254	6.16E-11	0.485573398	0.035493668	0.834717513	1.32E-05
ILMN_1717381	2q31.1	HOXD1	1.149018854	0.001869334	−0.016326993	0.979370587	1.257530218	0.010351204
ILMN_1742330	3p21.31	PLXNB1	1.151634332	4.77E-09	0.307456076	0.156975198	0.986064207	3.14E-06
ILMN_1765574	6p24	TFAP2A	1.155987834	3.47E-06	0.894639838	0.00485557	0.64083042	0.021720434
ILMN_1808837	9q34	RPL7A	1.157624855	4.61E-09	0.387443005	0.109745518	0.794960725	0.002820649
ILMN_3243291	12q13.1	HNRNPA1	1.168422696	5.16E-08	0.379313703	0.339819137	0.794206907	0.00808425
ILMN_3294222	7p15.3	RPS2P32	1.184870421	8.70E-06	0.512576125	0.231290236	0.670509356	0.09278963

(*Continued*)

Probe	Map	Gene Symbol	LogFC.1 vs 2	Adj. *p*-value 1 vs 2	LogFC.2A vs 2B	Adj. *p*-value 2A vs 2B	LogFC.1 vs 2A	Adj. *p*-value 1 vs 2A
ILMN_3274596	3q28	EIF4A2	1.185844732	1.68E-08	0.081121203	0.881125075	1.117905095	2.20E-05
ILMN_2181892	Xq22	BEX2	1.18895458	3.01E-07	−0.000435374	0.998680003	1.163167865	8.68E-05
ILMN_1758049	1p31.3-p31.2	NFIA	1.196694805	5.29E-11	0.129687319	0.120214425	1.088415295	2.55E-08
ILMN_1793990	2p25	ID2	1.204169896	3.69E-07	0.119990511	0.723833155	1.235304813	4.16E-06
ILMN_1672503	8p22-p21	DPYSL2	1.204618695	5.30E-06	0.308264815	0.574172971	1.094493565	0.000820227
ILMN_3243616	20q13.33	LOC100127888	1.216677914	5.32E-06	0.665181424	0.054251792	0.715201734	0.061915381
ILMN_3309349	4q26	SNHG8	1.217538898	0.000687661	0.283230754	0.711384187	0.981324303	0.048967414
ILMN_1775743	12q22	BTG1	1.227172139	2.34E-07	0.27512776	0.542971946	1.06785611	0.000259036
ILMN_1732154	1q31	BCAN	1.234925957	0.000329621	0.092682046	0.859477273	1.23256803	0.005531251
ILMN_2146389	5q33	MEGF10	1.244835792	1.36E-10	0.018019419	0.80518906	1.199536868	2.11E-07
ILMN_1713529	5q23.1	SEMA6A	1.245867424	5.97E-06	1.073177232	0.001992219	0.593865104	0.056583351
ILMN_1690342	12q22	LTA4H	1.247267616	2.31E-12	0.279998099	0.14712732	1.09837339	1.85E-09
ILMN_1684982	7q21.3	PDK4	1.272845665	1.11E-05	0.096661757	0.689596535	1.098202322	0.006094742
ILMN_1655422	18q21	RPL17	1.278107874	5.41E-08	−0.07032434	0.910619571	1.349116661	1.47E-05
ILMN_1685917	4q24	EMCN	1.278180642	1.09E-07	0.056026727	0.915318731	1.212684394	0.000370253
ILMN_1751904	13q22	EDNRB	1.280184833	0.000167768	0.960543451	0.048334041	0.739750872	0.09122889
ILMN_1740234	10q25.1	GSTO2	1.289190898	1.75E-05	0.093163282	0.796473023	1.151420901	0.003569825
ILMN_1658498	1p21.3	SLC44A3	1.291554929	6.26E-09	0.461201737	0.037553517	0.813982711	0.003691123
ILMN_1716014	3p24.2	RPL15	1.294050275	8.81E-09	0.135250391	0.780674971	1.208660273	1.11E-05
ILMN_3287068	9q21.13	RPSAP9	1.30214539	1.73E-10	0.188443385	0.505045669	1.218323469	1.07E-07
ILMN_1692058	15q11.2-q12	NDN	1.30284313	2.29E-06	0.032000201	0.974399337	1.299779461	0.0002229
ILMN_1790881	2q22.1	HNMT	1.305607283	1.12E-08	0.243631764	0.457196304	1.187279893	6.88E-06
ILMN_1754969	3p26-p24	LMCD1	1.313718501	4.86E-11	0.016442545	0.910393484	1.346172489	1.85E-09
ILMN_1733305	3q25.1	EIF2A	1.315492384	3.32E-14	0.313502771	0.03359472	1.070634923	6.06E-08
ILMN_1785424	10q25	ABLIM1	1.319568054	1.30E-05	0.372574362	0.166192781	1.14573872	0.002051582
ILMN_1671554	2p25.1	LPIN1	1.320195822	4.10E-10	0.360134813	0.129333387	1.065648353	6.28E-06
ILMN_1691053	16p13.3	RPS2	1.337410651	6.42E-09	0.245972769	0.453571352	1.092622198	0.000327466
ILMN_1772627	4p16.3	NSG1	1.339033154	4.83E-08	0.41835911	0.179117132	1.140256143	0.000119501
ILMN_1682459	19p13.3	TUBB4A	1.351559415	3.03E-07	0.738871781	0.006675471	0.965868934	0.000481317
ILMN_3227023	9q34.3	SNHG7	1.365658127	2.11E-08	0.806325225	0.056357312	0.858783102	0.003469886
ILMN_1679324	3p22.1	EIF1B	1.399790804	7.99E-11	−0.095756672	0.831345998	1.444967125	2.93E-08
ILMN_1712786	7q32.1	AHCYL2	1.406766726	7.62E-07	0.35055169	0.490113011	1.162861731	0.00092869
ILMN_1736670	10q23-q24	PPP1R3C	1.41282522	1.45E-05	0.96377997	0.002052965	0.79295205	0.051301141
ILMN_3289100	3p21.3-p21.2	RPL29	1.424227229	5.53E-10	0.407122491	0.223840114	1.151857458	1.92E-05
ILMN_2374115	6p24	TFAP2A	1.427376215	4.22E-09	0.628374495	0.005203369	0.993976042	0.000183433
ILMN_2218935	7q31	GPR37	1.454469504	2.02E-05	0.872541243	0.035064285	0.739604938	0.124983922
ILMN_3233229	9q34.3	SNHG7	1.461632818	4.77E-08	0.759171839	0.078824777	0.92004441	0.006157917
ILMN_1807050	15q21.1-q21.2	SHC4	1.463334644	8.75E-09	0.020460022	0.925436808	1.488818569	8.90E-07
ILMN_1687978	12q15	PHLDA1	1.507837374	3.37E-09	0.511392248	0.067518294	1.309639231	5.43E-07
ILMN_2176592	3q26.1-q26.2	BCHE	1.510293282	4.95E-11	0.134085017	0.602893484	1.261652892	1.37E-05

(*Continued*)

Probe	Map	Gene Symbol	LogFC.1 vs 2	Adj. *p*-value 1 vs 2	LogFC.2A vs 2B	Adj. *p*-value 2A vs 2B	LogFC.1 vs 2A	Adj. *p*-value 1 vs 2A
ILMN_3226291	3p21.3-p21.2	RPL29	1.518659314	1.91E-10	0.481222714	0.132924077	1.184637718	1.32E-05
ILMN_1751346	12q13	ERBB3	1.533761351	3.27E-08	0.825608838	0.025235833	0.909270118	0.005950394
ILMN_1701933	4q21	SNCA	1.540994797	4.27E-06	0.511236896	0.354547349	1.266961361	0.00231439
ILMN_1659550	12q14.3	RPSAP52	1.626872445	2.02E-07	0.077636637	0.933596867	1.580409429	6.17E-05
ILMN_3219764	3p22.2	RPSA	1.632917134	4.86E-11	0.316400233	0.404253808	1.482346609	1.34E-07
ILMN_1781758	3q21.2	ROPN1B	1.636828911	6.44E-11	0.078094634	0.763868135	1.595439593	5.09E-08
ILMN_1780799	8q24.1	ENPP2	1.707714414	5.06E-06	0.327647641	0.395114959	1.680884421	0.000104187
ILMN_2373791	8q24.1	ENPP2	1.707933561	6.62E-06	0.358956114	0.354138992	1.649444827	0.000181608
ILMN_1741404	8q21	MSC	1.713599687	2.22E-06	−0.045935557	0.850037685	1.982108139	8.39E-06
ILMN_3268403	19q13.43	LOC100128252	1.73596967	2.65E-15	0.063595835	0.808209561	1.607147123	8.90E-11
ILMN_1726460	3p22-p21.2	RPL14	1.810952692	2.66E-13	0.420072629	0.104764819	1.442303419	1.34E-07
ILMN_1703572	13q21	PCDH20	1.814353409	1.06E-06	0.178842659	0.464299956	1.481972865	0.004839451
ILMN_3307906	1p22-p21	PALMD	1.961562592	3.74E-08	−0.006321993	0.968956764	2.054763961	1.24E-06
ILMN_2344120	3q21.2	ROPN1B	1.966947982	5.29E-11	0.202607827	0.553888801	1.843453117	7.49E-08
ILMN_1668766	3q21.1	ROPN1	2.0412616	4.86E-11	0.156561029	0.632441945	1.953367657	2.93E-08
ILMN_2166457	4q34-q35	HPGD	2.082430064	4.76E-10	0.007126048	0.967369229	2.047051746	6.91E-07
ILMN_1733860	3p14.2	SYNPR	2.295113339	1.43E-07	0.418079902	0.312398369	2.034535612	5.53E-05
ILMN_1749875	12p13.31	LOC728715	2.403788128	7.54E-08	1.172083628	0.006374597	1.622884723	0.001103957
ILMN_2374449	4q22.1	SPP1	2.557887027	3.52E-05	−0.560528642	0.432466398	3.204139236	3.00E-05
ILMN_1651354	4q22.1	SPP1	2.607482094	7.67E-05	−0.709302937	0.426138115	3.293710762	7.04E-05

Supplementary Table S3: Distribution of chromosomal imbalances over the three expression classes

Aberration	Total (64)	Class I (25)	Class IIa (14)	Class IIb (25)	Class I vs. II	Class I vs. IIa	Class IIa vs. IIb	Class I vs IIb
8q								
-Normal	14	13	1	0	$p < 0.0001$	$p = 0.005$	$p = 0.148$*	$p < 0.0001$
-Trisomy	23	8	7	8	$p = 0.001$	$p = 0.013$*	$p = 0.228$*	$p = 0.001$*
-Amplification	27	4	6	17	$p = 0.001$	$p = 0.069$*	$p = 0.126$	$p = 0.0002$
Monosomy 3	41	3	13	25	$p < 0.0001$	$p < 0.0001$	$p = 0.148$	$p < 0.0001$
Gain 6p	43	23	8	12	$p = 0.001$	$p = 0.01$*	$p = 0.584$	$p = 0.001$
Loss 1p36	37	15	8	14	$p = 0.777$	$p = 0.862$	$p = 0.945$	$p = 0.774$
Loss 16q	21	9	2	10	$p = 0.664$	$p = 0.134$*	$p = 0.083$*	$p = 0.771$

*Likelihood ratio's
